# Mobile Digital Education for Health Professions: Systematic Review and Meta-Analysis by the Digital Health Education Collaboration

**DOI:** 10.2196/12937

**Published:** 2019-02-12

**Authors:** Gerard Dunleavy, Charoula Konstantia Nikolaou, Sokratis Nifakos, Rifat Atun, Gloria Chun Yi Law, Lorainne Tudor Car

**Affiliations:** 1 Centre for Population Health Sciences Lee Kong Chian School of Medicine Nanyang Technological University Singapore Singapore Singapore; 2 Graduate School of Medicine The University of Tokyo Tokyo Japan; 3 Health Informatics Centre Karolinska Institutet Stockholm Sweden; 4 Harvard TH Chan School of Public Health Harvard University Boston, MA United States; 5 Harvard Medical School Harvard University Boston, MA United States; 6 Family Medicine and Primary Care Lee Kong Chian School of Medicine Nanyang Technological University Singapore Singapore Singapore; 7 Department of Primary Care and Public Health School of Public Health Imperial College London London United Kingdom

**Keywords:** mLearning, digital education, health workforce, systematic review, meta-analysis

## Abstract

**Background:**

There is a pressing need to implement efficient and cost-effective training to address the worldwide shortage of health professionals. Mobile digital education (mLearning) has been mooted as a potential solution to increase the delivery of health professions education as it offers the opportunity for wide access at low cost and flexibility with the portability of mobile devices. To better inform policy making, we need to determine the effectiveness of mLearning.

**Objective:**

The primary objective of this review was to evaluate the effectiveness of mLearning interventions for delivering health professions education in terms of learners’ knowledge, skills, attitudes, and satisfaction.

**Methods:**

We performed a systematic review of the effectiveness of mLearning in health professions education using standard Cochrane methodology. We searched 7 major bibliographic databases from January 1990 to August 2017 and included randomized controlled trials (RCTs) or cluster RCTs.

**Results:**

A total of 29 studies, including 3175 learners, met the inclusion criteria. A total of 25 studies were RCTs and 4 were cluster RCTs. Interventions comprised tablet or smartphone apps, personal digital assistants, basic mobile phones, iPods, and Moving Picture Experts Group-1 audio layer 3 player devices to deliver learning content. A total of 20 studies assessed knowledge (n=2469) and compared mLearning or blended learning to traditional learning or another form of digital education. The pooled estimate of studies favored mLearning over traditional learning for knowledge (standardized mean difference [SMD]=0.43, 95% CI 0.05-0.80, N=11 studies, low-quality evidence). There was no difference between blended learning and traditional learning for knowledge (SMD=0.20, 95% CI –0.47 to 0.86, N=6 studies, low-quality evidence). A total of 14 studies assessed skills (n=1097) and compared mLearning or blended learning to traditional learning or another form of digital education. The pooled estimate of studies favored mLearning (SMD=1.12, 95% CI 0.56-1.69, N=5 studies, moderate quality evidence) and blended learning (SMD=1.06, 95% CI 0.09-2.03, N=7 studies, low-quality evidence) over traditional learning for skills. A total of 5 and 4 studies assessed attitudes (n=440) and satisfaction (n=327), respectively, with inconclusive findings reported for each outcome. The risk of bias was judged as high in 16 studies.

**Conclusions:**

The evidence base suggests that mLearning is as effective as traditional learning or possibly more so. Although acknowledging the heterogeneity among the studies, this synthesis provides encouraging early evidence to strengthen efforts aimed at expanding health professions education using mobile devices in order to help tackle the global shortage of health professionals.

## Introduction

In 2013, the World Health Organization estimated that there was a shortage of 17.4 million health care workers worldwide: around 2.6 million doctors, and approximately 9 million nurses and midwives [1]. This shortage is more apparent in certain regions like Africa where there is an average of 1.9 health workers per 1000 population when 4.5 are needed to reach the health-related sustainable development goals [2]. This situation is further exacerbated with the migration of both students and fully qualified workers, either from rural to urban areas within a country or migration outside the country [3]. This dearth and disproportionate distribution of health workers worldwide [4] may be aggravated by the inadequacy of training programs (in terms of content, organization, and delivery) to provide trainees with the necessary skills, competencies, and experience for the context in which they will work [5]. Therefore, focused effort and resources are needed to develop and implement strategies aimed at increasing both the number of health professionals and the quality and relevance of their training [2,6]. The deployment of information and communication technologies for educational purposes (ie, digital education) has been recognized as a strategic platform to build robust health professions education and training systems [7].

Digital education is a broad construct describing a wide range of teaching and learning strategies that are exclusively based on the use of electronic media and devices as training, communication, and interaction tools [8]. The construct covers aspects that may pertain to educational approaches, concepts, methods and technologies [9]. Digital education facilitates distant learning, which may help address the shortage of health professionals and educators in settings with limited resources by reducing the constraints of time and geographic barriers to training. When digital education is used alongside traditional educational strategies such as classroom-based, face-to-face teaching, this method of education can be considered *blended learning*.

Digital education can entail various types of interventions that can be characterized in different ways: according to delivery tools, content, learning objectives, pedagogical approaches or settings of delivery. We categorized digital education according to the mode of delivery of digital education intervention and the pedagogical methods. Digital education includes, but is not limited to, offline and online computer-based education, serious gaming and gamification, massive open online courses, virtual reality environments, augmented reality, virtual patient simulations, psychomotor skills trainers and mobile digital education (mLearning) among others [9]. Each of these types of digital education has its own specificities, advantages, limitations, and challenges. This review is part of a series of reviews [10-14] evaluating the efficacy of different types of digital education in improving skills, knowledge, attitudes, and ultimately clinical competencies of pre and postregistration health professionals. This review focuses on mLearning for pre and postregistration health professions education [14].

There is no uniformly accepted definition of mLearning. This lack of consensus not only arises from the rapid evolution of the field but also from ambiguity of the term “mobile.” Earlier definitions of mLearning were technocentric and only focused on the types of devices used, (eg, through a smartphone or tablet), or the situational context in which learning takes place (eg, on the way back home) [15], whereas more recent definitions of mLearning give more weight to the learner and the context in which the learning takes place. In the Handbook of Mobile Learning, mLearning was defined as “learning across multiple contexts, through social and content interactions, using personal electronic devices” [16]. However, the latter definition creates ambiguity around the type of devices, particularly given the number of personal consumer devices, such as laptops, that are currently available in the market. To avoid such ambiguity, we considered mLearning in health professions education as any intervention using handheld, mobile devices connected through wireless connections to deliver educational content to pre and postregistration health professionals in order to extend the reach of learning and teaching beyond physical space and distance.

mLearning is increasingly used in health professions education before (preregistration) and after qualification (postregistration), for example, as part of specialty training, continuous medical education or continuous personal development. In this review, we present the evidence collated on the use of mLearning in pre and postregistration health professions education. We considered eligible studies on candidates for, and holders of, the qualifications listed in the Health Field of Education and Training of the International Standard Classification of Education. We combine both the technocentric and the learner-centered approaches by defining handheld, mobile devices as being “small, autonomous, and unobtrusive enough to accompany us in every moment of our every-day life” [17]. Arguably, considering the recent advances in the capabilities of modern handheld devices, many if not all of the digital education interventions could foreseeably be delivered via mLearning.

Past reviews have underlined the potential of mLearning interventions but also stressed upon the need for further research and reviews on the topic [18-21]. Considering the rapidly evolving nature of mLearning technologies, up-to-date evidence is essential to evaluate the effectiveness of mLearning for health professions education. The most recently published of these reviews was in 2015 with a search strategy that was applied in 2012 [19]. However, the technology and field of mLearning have advanced rapidly since. The past reviews had methodological flaws, which as a result garnered less evidence, with some reviews focusing singularly on 1 medium of mLearning rather than being inclusive across a range of mLearning devices [20]. With a more robust and systematic methodology, this review collates new evidence published since these reviews were performed [18-21], providing a more comprehensive, focused and up-to-date evaluation of mLearning in health professions education.

The primary objective of this review is to evaluate the effectiveness of mLearning educational interventions for delivering preregistration and postregistration health professions education.

## Methods

We adhered to the published protocol [14] and followed Cochrane methodology in every step of the review [22]. For a more detailed description of the methodology, please refer to the methodology paper by Car J et al [9].

### Search Strategy and Data Sources

#### Electronic Searches

We developed a comprehensive search strategy for Medical Literature Analysis and Retrieval System Online (Ovid), EMBASE (Elsevier), Cochrane Central Register of Controlled Trials (Wiley), PsycINFO (Ovid), Educational Research Information Centre (Ovid), Cumulative Index to Nursing and Allied Health Literature (Ebsco) and Web of Science Core Collection (Thomson Reuters). Multimedia Appendix 1 contains the Medical Literature Analysis and Retrieval System Online [Ovid] search strategy used. Databases were searched from January 1990 to August 2017. The reason for selecting 1990 as the starting year for our search is because before this year, the use of mobile devices for education was limited to very basic tasks. There were no language restrictions.

We searched reference lists of all the studies that we deemed eligible for inclusion in our review and relevant systematic reviews. We also searched the International Clinical Trials Registry Platform Search Portal and metaRegister of Controlled Trials to identify unpublished trials from and including 1990 to August 16, 2017.

#### Inclusion Criteria

We included RCTs and cluster RCTs. We excluded crossover trials because of a high likelihood of a carry-over effect. We included studies with students enrolled in either preregistration or postregistration health professions educational programs. We defined preregistration, undergraduate education or basic vocational training as any type of study leading to a qualification that (1) is recognized by the relevant governmental or professional bodies of the country where the studies were conducted and (2) entitles the qualification-holder to apply for entry-level positions in the health care workforce. Postregistration health profession educational programs were defined as any type of study after a qualification, which is recognized by the relevant governmental or professional bodies that enable the qualification holder entry into or continuation of work in the health care workforce in a more independent or senior role. Participants were not excluded based on age, gender, or any other sociodemographic characteristic.

We included studies in which mLearning interventions were used to deliver the learning content of the course. This includes studies where mLearning methods were the sole means by which the intervention was delivered or where mLearning methods were used in combination with traditional learning (ie, blended learning), as long as the contribution of the mLearning component to overall learning has been assessed.

mLearning interventions were defined as any teaching, learning and/or training intervention that was delivered through handheld mobile devices using wireless transmissions: third generation of mobile telecommunications technology, fourth generation of mobile telecommunications technology, global system for mobile communications, originally groupe spécial mobile (GSM), general packet radio services (GPRS), enhanced data rates for GSM evolution (EDGE or EGPRS), multimedia messaging service, short message service, universal mobile telecommunications system, wireless networking (Wi-Fi or any other wireless local area network) or long term evolution standard. Handheld mobile devices include but are not limited to mobile phones, smartphones, personal digital assistants (PDAs), tablets and Moving Picture Experts Group-1 audio layer 3 (MP3) players.

To be eligible for inclusion, studies have to report at least one of the following primary or secondary outcomes. The primary outcomes (measured using any validated or nonvalidated instruments) were the following: (1) students’ knowledge postintervention scores, (2) students’ skills postintervention, (3) students’ attitudes postintervention toward the mLearning intervention, education, or new clinical knowledge, and (4) learners’ satisfaction postintervention with the mLearning intervention.

Secondary outcomes were patient-related outcomes, change in clinical practices, economic aspects of the mLearning interventions (eg, cost-effectiveness, implementation cost, return on investment), changes in accessibility and/or availability of education and any adverse and/or unintended effects of mLearning interventions.

### Data Collection and Analysis

#### Selection of Studies

The search results from different electronic databases were combined in a single library and duplicates were removed. A total of 2 authors (GD and CKN) independently screened titles and abstracts of all records to identify potentially relevant studies. We retrieved full-text copies of those articles deemed potentially relevant. Finally, the same 2 reviewers independently assessed the full-text versions of the retrieved articles against the eligibility criteria. Any disagreements were resolved through discussion between the 2 screeners with a third review author (LTC) acting as an arbiter.

#### Data Extraction and Management

A total of 2 reviewers out of 4 (GD, CKN, LTC, and SN) independently extracted relevant characteristics related to participants, intervention, comparators, outcome measures, and results from all the included studies using a standard data collection form (see Multimedia Appendix 2). Any disagreements were resolved by discussion. We contacted study authors for any missing information.

#### Assessment of Risk of Bias in Included Studies

A total of 2 authors out of 3 (GD, CKN, and LTC) independently assessed the risk of bias of RCTs and cluster-RCTs using the Cochrane risk of bias assessment tool [22]. We piloted the risk of bias assessment among the reviewers and contacted study authors in case of any unclear or missing information. We assessed risk of bias in included RCTs using the following domains: random sequence generation, allocation sequence concealment, blinding (participants, personnel), blinding (outcome assessment), completeness of outcome data, selective outcome reporting, and other sources of bias (eg, baseline imbalance, inappropriate administration of an intervention, and contamination).

For cluster RCTs, we also assessed the risk of these additional biases: recruitment bias, baseline imbalance, loss of clusters, incorrect analysis, and comparability with individually randomized trials. Judgments concerning the risk of bias for each study will be classified using “yes,” “no,” or “unclear,” indicating high, low, or unclear risk of bias, respectively. We incorporated the results of the risk of bias assessment into the review using a graph and a narrative summary.

#### Measures of Treatment Effect

We were unable to identify a clinically meaningful interpretation of effect size in the literature for digital education interventions. Therefore, in line with other studies in the field, we present outcomes using postintervention standardized mean difference (SMD) and interpret the effect size using Cohen’s ‘rule of thumb’ (ie, with 0.2 representing a small effect, 0.5 a moderate effect, and 0.8 a large effect) [22,23]. This type of effect size interpretation has been used in previous studies [23]. For papers that reported median and range for the various outcomes, we converted this to mean and SD via the method mentioned by Wan et al [24], and then recalculated these values to provide an SMD for each outcome measure. We used the standard way to convert the results as recommended by Cochrane [22].

#### Data Synthesis

We aimed to present uniform postintervention data (ie, SMDs for continuous outcomes with their respective confidence intervals) to ensure consistency and comparability of data. For the meta-analysis, we used a random-effects model as different scales were used in different studies. We used the generic inverse variance method to combine cluster and noncluster RCTs of continuous outcomes. The effect estimates with corresponding 95% CIs for each study as well as a pooled effect size with 95% CI were displayed in the forest plots. We performed meta-analysis using Review Manager 5.3 (Cochrane Library Software, Oxford, UK) [25]. We adhered to the statistical approach described in the Cochrane Handbook [22].

We developed a preliminary synthesis by grouping the included studies by the type of interventions and comparators used:

1. mLearning versus traditional learning
2. Blended learning versus traditional learning
3. mLearning versus other forms of digital education

We prepared a *Summary of Findings* table to present a summary of the results and a judgment on the quality of the evidence, on the basis of the methods described in chapter 11 of the Cochrane Handbook for Systematic Reviews of Interventions [26]. Moreover, 2 authors used the Grading of Recommendations, Assessment, Development and Evaluation (GRADE) criteria to rank the quality of the evidence using the GRADE profiler (GRADEpro) software [26].

## Results

### Results of the Search

**Figure 1:**
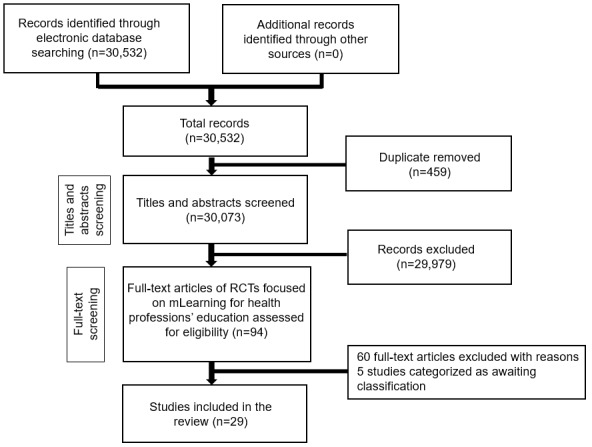
Study flow diagram. RCT: randomized controlled trial.

Our search strategy retrieved 30,532 unique references, of these, 29 studies fulfilled inclusion criteria [27-55] (see Figure 1).

### Included Studies

We included 29 studies involving 3175 participants [27-55] (see Multimedia Appendix 3 for characteristics of included studies).

**Figure 2:**
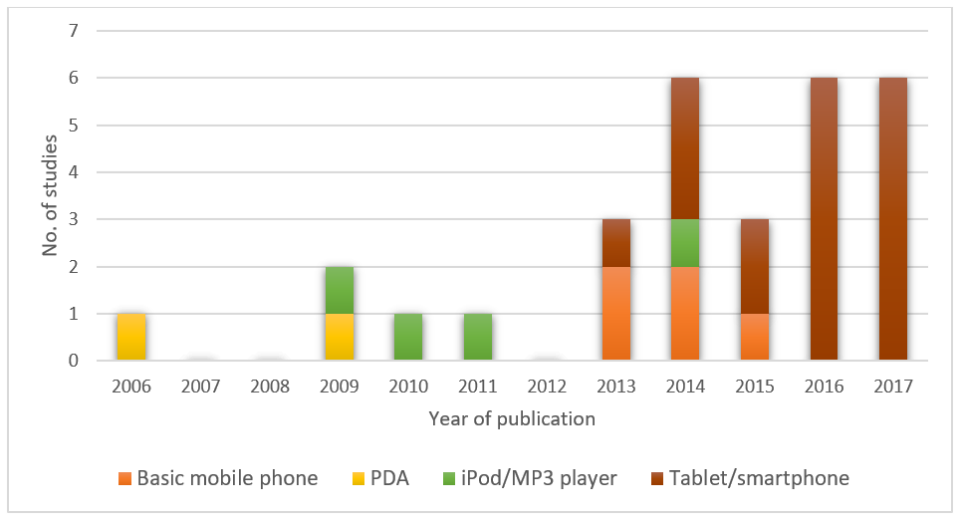
Number of studies by year of publication and mLearning device. PDA: personal digital assistant.

**Figure 3:**
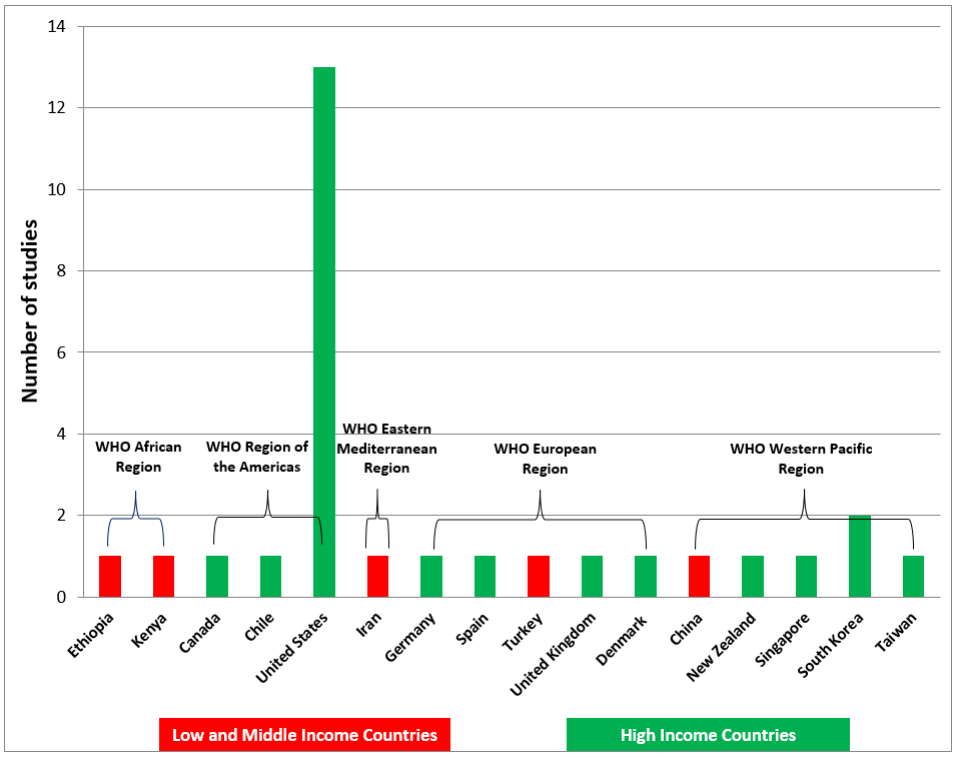
Country of origin of included studies for low- and middle-income and high-income countries separately. WHO: World Health Organization.

A total of 25 out of 29 studies were RCTs, and the remaining 4 studies were cluster RCTs [31,32,43,47]. A total of 26 studies randomized participants into 2 groups [28-39,41-45,47-55]. Furthermore, 1 study randomized participants into 3 groups [46] and 2 studies randomized participants into 4 groups [27,40]. Participants included preregistration and postregistration health professionals. A total of 15 studies included preregistration participants, 9 involving medical students [27,29,34,38, 42,44,45,48,50], 4 studies involved nursing students [33, 40,41,52], 1 study each, involved dental [37] and physiotherapy students [36]. A total of 13 studies included postregistration health professionals [27,31,32,35,39,43,46,47,49,51,53-55]. The postregistration health professional participants included registered nurses, physicians, internal medicine residents, family medicine residents, neurosurgeon trainees, midwives, health extension workers, and trauma and critical care fellows. One study involved pre and postregistration health professionals [30] (ie, medical students and gastroenterology residents and fellows). A total of 24 studies were conducted in high-income countries [27,29-31,33-41,44-49,51-55], 13 of which were conducted in the United States. A total of 4 studies were conducted in middle-income countries, including China [32], Iran [28], Kenya [50], and Turkey [42]. Only 1 study was conducted in a low-income country [43], namely Ethiopia. No included study was published before 2006. A total of 8 studies were published between 2006 and 2013 [33,34,38,40, 44,46,53,55], whereas the remaining 21 studies (72%) were published between 2014 and 2017 (see Figures 2 and 3). For the intervention groups, 18 studies used a tablet or smartphone device to deliver the intervention [27,29-31,34,36, 37,39,41-43,45,48-52,54]; 5 studies used basic mobile phones [28,32,33,44,47]; 3 studies used iPods [38,40,53]; 2 studies used a personal digital assistant [46,55], and 1 study used MP3 players [35] (see Figure 2). Only 6 studies directly mentioned the use of learning theories in their instructional design for mobile learning [33,37,38,40,42,51], each of which used theories that are of cognitive-behaviorist pedagogy [56]. A total of 2 studies adopted the cognitive theory of multimedia to improve clinical skills [38] or knowledge [42]; 3 studies adopted cognitive learning theories such as information processing theory [33], dual coding theory [37], and adult learning theory [51], and 1 study combined cognitive theory (ie, Bloom’s Taxonomy) with social constructivism [40]. The remaining 23 studies did not mention any learning theories explicitly in their reports. Most of the studies only described the teaching or instructional practices in mLearning, which lead to the change of knowledge, skills, attitudes, or satisfaction. For the control groups in the included studies, 26 studies used traditional forms of learning (eg, didactic lectures, conference, small group teaching, paper-based, standard box trainer, clinical placement, or usual learning) [28,30-45,47-55]; 2 studies used a different form of mLearning intervention (eg, limited functions) [27,46]; 1 study used another form of digital education (eg, video access to a lecture) [29]. The duration of the interventions ranged from 20 min [39] to 12 months [43,51]. One study did not report the duration of the intervention [40]. There were no studies reporting repeated interventions.

**Figure 4:**
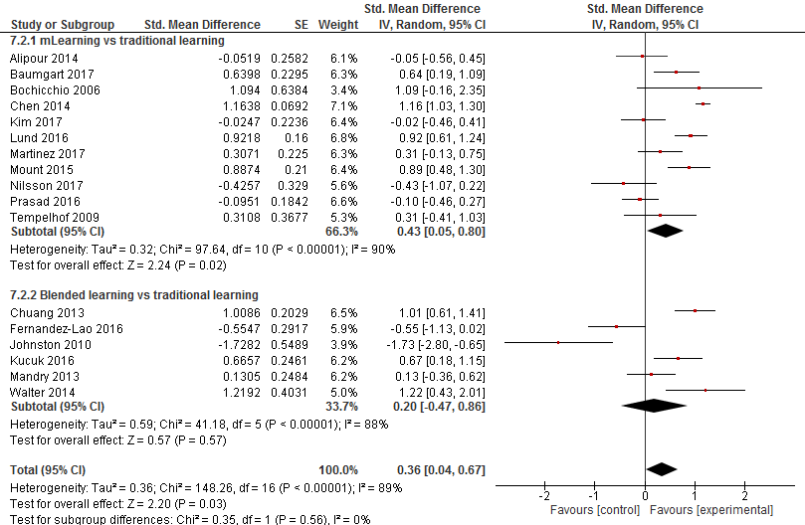
Forest plot for the knowledge outcome (postintervention).

### Primary Outcomes

#### Knowledge

A total of 20 studies (n=2469) assessed knowledge posttest scores as a primary outcome [27-30,32,33,36,40-47,49,51,53-55] with 75% of studies using multiple choice questionnaires (MCQs) as their outcome measure. A total of 13 studies assessed knowledge using nonvalidated instruments [27-29,32,36,40, 43,45,49,51,53-55]. A total of 5 studies stated measures that were performed to validate their measurement instruments [33,41,42,46,47], whereas 2 studies stated using standardized tests that are regularly used in medical education [30,44]. A total of 17 studies assessed knowledge posttest scores as a primary outcome immediately postintervention [27-30,32,36, 40,42-46,49,51,53-55]; 1 study assessed knowledge 1 week postintervention [33]; 1 study assessed knowledge 1 month postintervention [41]; 1 study assessed knowledge 6 weeks postintervention [47]. A total of 10 studies focused on postregistration health professions education [28,32,43,46, 47,49,51,53-55]. A total of 9 studies focused on preregistration health professions education [27,29,33,36,40-42,44,45], whereas the remaining 1 study included both pre and postregistration health professions education [30].

##### mLearning Versus Traditional Learning

A total of 11 studies compared mLearning methods versus traditional learning, assessing knowledge gain postintervention [28,30,32,41,43,45,47,49,51,53,55] (n=1828). For a summary of the effects of these comparisons on knowledge scores, see Multimedia Appendix 4.

The pooled estimate of the studies favored mLearning over traditional learning in terms of postintervention knowledge scores (SMD=0.43, 95% CI 0.05-0.80, N=11 studies, low-quality evidence; see Figure 4). There was a substantial amount of heterogeneity in the pooled analyses (I^2^=90%).

##### Blended Learning Versus Traditional Learning

A total of 6 studies compared blended learning methods (mLearning plus traditional learning) with traditional learning to assess knowledge gain postintervention [33,36,40,42,44,54] (n=345). For a summary of the effects of these comparisons on knowledge scores, see Multimedia Appendix 5.

There was no difference between blended learning and traditional learning groups in terms of postintervention knowledge scores (SMD=0.20, 95% CI –0.47, 0.86, N=6 studies, low-quality evidence; see Figure 4). There was a substantial amount of heterogeneity in the pooled analyses (I^2^=88%).

##### mLearning Versus mLearning

A total of 2 studies compared one form of mLearning with another form of mLearning to assess knowledge gain postintervention [27,46]. Of these, 1 study (63 participants) included 4 groups receiving varying forms of an mLearning intervention, viewing an iPad with a podcast that was either a narrated presentation for group 1, a narration with video demonstration of skills for group 2, a narrated presentation with guided mental practice for group 3, or a narrated presentation with video demonstration of skill and guided mental practice for group 4 [27]. Knowledge gain was significantly higher for both group 2 and group 3 compared with group 1 (*P*=.01; *P*=.01, respectively); knowledge gain was also significantly higher for group 4 compared with all other groups. Furthermore, 1 study (72 participants) included 3 groups; however, only 2 of these were randomized [46]. The 2 randomized groups (38 participants) received either a basic PDA or a PDA with an additional software, a Geriatric Assessment Tool (GAT) program added. The authors reported that the PDA with additional GAT software may have little or no difference in knowledge gain postintervention compared with the basic PDA group (SMD=0.03, 95% CI –0.61 to 0.67, small effect size) [46].

##### mLearning Versus Another Form of Digital Education

One study (100 participants) compared mLearning with another form of digital education to assess knowledge gain postintervention [29]. The study compared an mLearning group who used the module Carpal Tunnel Surgery on the Touch Surgery app 3 times with a group who watched an audio-dubbed slide show lecture 3 times [29]. Compared with another form of digital education, mLearning was reported to improve postintervention knowledge (SMD=1.82, 95% CI 1.35-2.29, large effect size) [29].

#### Skills

A total of 14 studies assessed skill acquisition of mLearning interventions compared with various controls and included a total of 1097 participants [27,31,34-39,41,43,48-50,52]. A total of 11 studies used direct observation assessments to assess skills [27,31,34,36-39,41,43,49,50], 1 study used a timed quiz [48], 1 study used a survey [52], and 1 study used an MCQ to assess skills [35]. A total of 8 studies assessed cognitive skills [27,35,36,41,43,48-50], while 4 studies assessed psychomotor skills [34,37-39] and further 2 studies assessed nontechnical skills [31,52]. All 14 studies that assessed skills assessed the outcome immediately postintervention. A total of 9 studies focused on preregistration health professionals [27,34,36-38,41,48,50,52]. A total of 5 studies focused on postregistration health professionals [31,35,39,43,49].

##### mLearning Versus Traditional Learning

A total of 5 studies compared mLearning methods with traditional learning, assessing skill acquisition postintervention [31,35,41,43,49] (n=529). For a summary of the effects of these comparisons on skill acquisition scores, see Multimedia Appendix 4.

**Figure 5:**
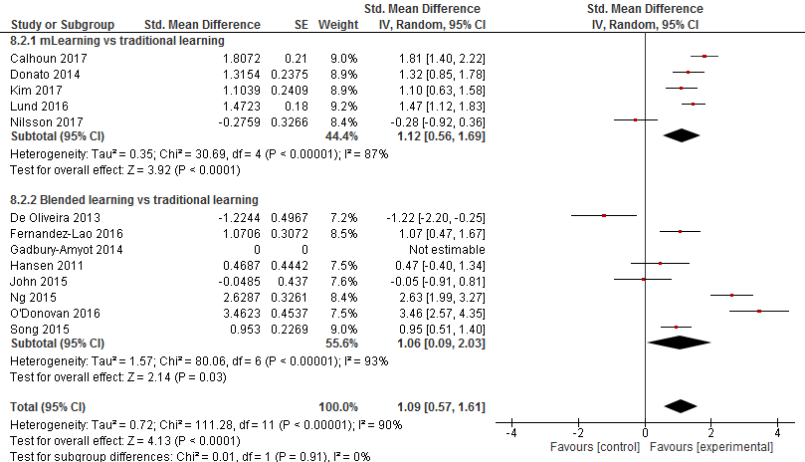
Forest plot for the skills outcome (postintervention).

The pooled estimate of the studies favored mLearning over traditional learning in terms of postintervention skill acquisition (SMD=1.12, 95% CI 0.56 to 1.69, N=5 studies, moderate quality evidence; see Figure 5). There was a substantial amount of heterogeneity in the pooled analyses (I^2^=87%).

##### Blended Learning Versus Traditional Learning

A total of 8 studies compared blended learning methods versus traditional learning, assessing skill acquisition postintervention [34,36-39,48,50,52] (n=504). For a summary of the effects of these comparisons on knowledge scores, see Multimedia Appendix 5.

The pooled estimate of the studies favored blended learning over traditional learning in terms of postintervention skill acquisition scores (SMD=1.06, 95% CI 0.09-2.03, N=7 studies, low-quality evidence; see Figure 5). There was a substantial amount of heterogeneity in the pooled analyses (I^2^=93%).

We are uncertain about the effect of 1 study (183 participants) because of incomparable outcome data [37]. However, the authors reported that blended learning may have little or no difference in dental procedural skill acquisition at postintervention compared with traditional learning [37].

##### mLearning Versus mLearning

One study (63 participants) included 4 groups receiving varying forms of an mLearning intervention, as was described earlier in the review [27]. Skill acquisition was assessed using a key-elements scale, critical error checklist, and the Ottawa global rating scale (GRS), as students had to manage a manikin-based simulated airway crisis. Group 1 significantly underperformed in comparison with all other groups on the key-events scale, the critical error checklist, and the Ottawa GRS. Group 4 reported greater improvement on the key events checklist compared with group 2 and 3, but there was no difference in terms of the critical error checklist and the Ottawa GRS between the groups.

#### Attitude

A total of 5 studies assessed participants’ attitudes following the mLearning or blended learning intervention and included a total of 440 participants [35,37,38,41,48]. All studies assessing attitude used self-report surveys. All studies assessing attitude, assessed attitude in terms of the participants’ self-confidence as a result of the intervention. A total of 4 studies focused on preregistration health professionals [37,38,41,48], whereas 1 study focused on postregistration health professionals [35].

##### mLearning Versus Traditional Learning

A total of 2 studies comparing mLearning methods versus traditional learning assessed participants’ attitude postintervention [35,41] (n=167). For a summary of the effects of these comparisons on attitudes, see Multimedia Appendix 4.

The pooled estimate of the studies favored mLearning over traditional learning in terms of postintervention attitudes (SMD=0.51, 95% CI 0.20-0.81, N=2 studies, low-quality evidence, I^2^=0%).

##### Blended Learning Versus Traditional Learning

One study (72 participants) reported that participants in the blended learning group felt more confident in their ability to identify the anatomical structures postintervention compared with traditional learning [48]. Furthermore, 1 study (21 participants) assessed learners’ postintervention attitude and reported inconclusive findings in terms of postintervention self-confidence for both male and female catheterization [38]. A further study (183 participants) assessed participants’ post-intervention attitudes toward their intervention, but this was only conducted among the blended learning intervention group; therefore, we were unable to judge the effect of the interventions [37].

#### Satisfaction

A total of 4 studies [33,35,36,41] assessed participants’ satisfaction following mLearning or blended learning interventions compared with various controls and included a total of 327 participants. A total of 2 studies assessed satisfaction with the learning method in both the intervention and control groups [35,41], whereas the remaining 2 studies only assessed satisfaction with the learning method in the intervention group [33,36]. A total of 3 studies focused on preregistration health professionals [33,36,41], whereas 1 study focused on postregistration health professionals [35].

##### mLearning Versus Traditional Learning

A total of 2 studies assessed participants’ postintervention satisfaction scores in the mLearning interventions compared with traditional learning [35,41] (n=167).

There was no difference between mLearning and traditional learning groups in terms of postintervention satisfaction (SMD=0.39, 95% CI –0.29, 1.06, N=2 studies, very low-quality evidence). There was a substantial amount of heterogeneity in the pooled analyses (I^2^=79%).

##### Blended Learning Versus Traditional Learning

No study assessed participants’ postintervention satisfaction scores in both the blended learning intervention group and the traditional learning group. A total of 2 studies [33,36] assessed participants’ post-intervention satisfaction in the blended learning intervention group only; therefore, we were unable to judge the effect of the interventions because of missing or incomparable outcome data.

### Secondary Outcomes

#### Cost-Effectiveness

A total of 2 studies [32,49] performed an economic analysis of mLearning interventions compared with traditional learning interventions. One study performed a more thorough and comprehensive economic comparison [49]. The Programme Effectiveness and Cost Generalization model for conducting cost-effectiveness analyses [57] was used to compare the mLearning group using a mobile app with the traditional learning group using textbooks. An incremental cost-effectiveness ratio of –861.967 (95 % CI –1071.7 to –3.2) US $/pct. point change in Objective Structured Assessment of Ultrasound Skills scale score was reported indicating that traditional learning was significantly more cost-effective than the mLearning [49]. In contrast, Chen et al 2014 reported that mLearning was more cost-effective than traditional learning [32]. Short message service (SMS) text messages over 6 weeks for the intervention group cost less than 2 Yuan (US $0.32) per health worker compared with 560 Yuan (US $89.96) per health worker for the 1-day training for the control group. An additional study reported on the cost of the mLearning intervention device used, namely the “connecTAB” [50]. Each “connecTAB,” which came preloaded with the intervention groups instructional videos, reportedly cost US $50.

#### Patient-Related Outcomes

One study reported on patient-related outcomes [43]. The primary outcome in the study was perinatal death, which was defined as a composite of a stillbirth or an early neonatal death. The mLearning intervention group, which included midwives and health extension workers, received a smartphone with the “Safe Delivery App” downloaded. The app included information and animated videos around the topic of perinatal survival. The control group engaged in standard care and did not receive an active intervention. A lower perinatal mortality of 14 per 1000 births was reported in the intervention clusters compared with 23 per 1000 births in control clusters; however, this difference was not significant. Similarly, the intervention group reported a lower stillbirth rate of 10 per 1000 births compared with 16 per 1000 births in control clusters, this difference was not statistically significant.

#### Changes in Clinical Practices/Behaviors

A total of 2 studies reported on changes in clinical practices/behaviors [32,55]. One study (n=479) reported on changes in antibiotic and steroid prescriptions comparing an mLearning group who received SMS text messages over a 6-week period with a traditional learning group who received standard continuous medical education [32]. In the mLearning group, there was no change in the prescription of antibiotics, whereas prescriptions for steroids fell by 5%. In contrast, for the traditional learning group, prescriptions for antibiotics and steroids increased by 17 and 11 percentage points, respectively. Antibiotic decision appropriateness was assessed in 1 study (n=12) but was only performed in the mLearning group; therefore, no comparison with the control group was possible [55]. The authors reported an improvement in antibiotic decision accuracy from 66% during the first 3 months to 86.6% during the second 3-month period. No other studies assessed secondary outcomes.

#### Adverse and/or Unintended Effects

None of the included studies reported any reported any adverse and/or unintended effects of the mLearning interventions.

#### Changes in Accessibility and/or Availability of Education

We were unable to assess the changes in accessibility and/or availability of education because of limited information in the included studies.

#### Sensitivity Analyses

There was not sufficient data to allow sensitivity analyses to be conducted.

#### Assessment of Publication Bias

There were not enough comparisons to carry out a formal assessment of publication bias.

#### Risk of Bias in Included Studies

**Figure 6:**
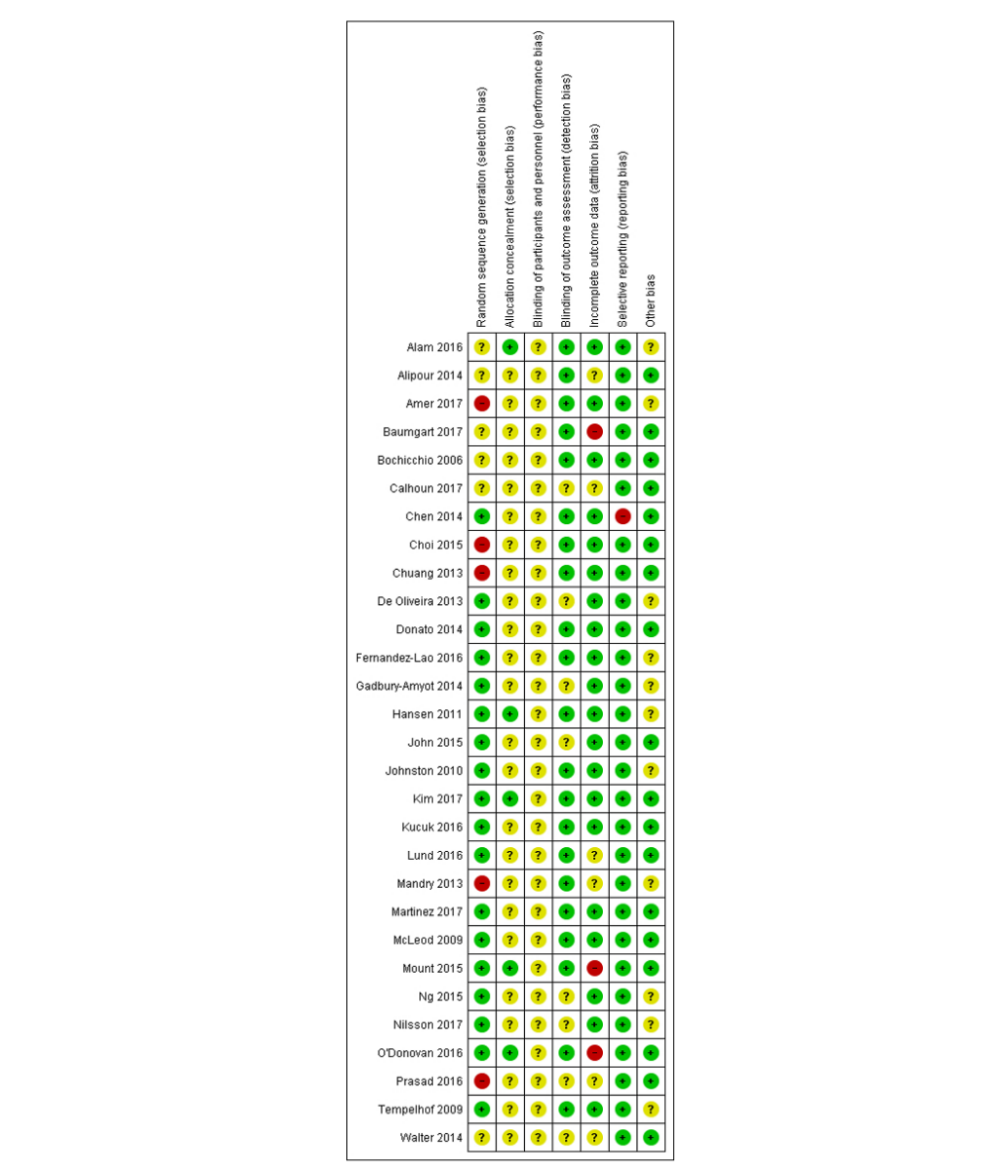
Risk of bias summary: review authors’ judgments about each risk of bias item across all included studies.

As presented in the risk of bias summary (Figure 6), the risk of bias was mostly judged to be high or unclear because of a lack of relevant information in the included studies (see Multimedia Appendix 6 for the risk of bias graph). We judged that the overall risk of bias was high in 16 studies (55%) as the studies had an unclear risk of bias in at least 4 out of 7 domains or a high risk in at least one domain [28-34,37,44,47-52,54]. We judged that the risk of bias was low in 2 studies (7%) because of the 2 components of selection bias being graded as low plus at least 3 of the remaining 5 domains [38,41].

More than four-fifth of studies (86%) did not provide information on the method of randomization and sequence allocation. The majority of studies (72%) reported the use of outcome measures to blind assessors or used self-report questionnaires or MCQs in the outcome assessment, which we believed did not require blinding, and thus these studies were judged to be of a low risk of bias. The remaining studies (28%) were judged to be of an unclear risk of bias because of a lack of information. A total of 3 studies (10%) were judged to be of a high risk of attrition bias as these studies had a high dropout rate (35%-73%) and/or no reasons for missing data were reported and/or lacked intention to treat analysis for the missing data, a further 6 studies (20%) were judged as unclear because of a lack of information. One study (3%) was judged to be of a high risk of bias for selective reporting, as an outcome stated in the methods section was not reported in the results section, the rest of the studies were judged to be of a low risk of bias for selective reporting. A total of 11 studies (38%) did not provide any information on a baseline assessment and were judged to be of an unclear risk of other bias.

In the clustered RCTs, only 1 study accounted for clustering reporting both individual level and cluster levels results [32], whereas there was no evidence of attrition of clusters in the studies. Additional analyses of the risk of bias for the cluster RCT are presented in Multimedia Appendix 7.

## Discussion

### Principal Findings

Learning is changing and is increasingly becoming mobile. Our findings suggest that mLearning interventions are equivalent or possibly superior to traditional learning methods for improving knowledge and skill in pre and postregistration health professions education (see the summary of findings Multimedia Appendices 4 and 5). Reporting that mLearning is as effective as traditional learning has important policy implications, and we do so with caution and consideration when interpreting these findings, acknowledging the high heterogeneity among the included studies. With more than 5 billion people owning a mobile phone worldwide [58], the global reach of mLearning is evident. mLearning’s potential to extend the reach of health professions training and education via mobile devices is significant.

With 21 of the 29 included studies (72%) published between 2014 and 2017, it’s clear that mLearning is an emerging educational strategy. The remaining 8 studies were published between 2006 and 2013, with no studies published before 2006, further highlighting the modern nature of this approach to health professions education and its relevance. The novel nature of mLearning, coupled with the continuing rapid advancements in mobile technology will likely see mLearning continue to evolve, as it has with the studies included in this review. This evolution is graphically illustrated in Figure 3, with PDAs being used more in the initial years, whereas for 14 out of the 15 studies from 2015 onwards, the main mode of delivery of mLearning was via smartphone/tablet devices. We identified a lack of research on mLearning in low- and middle-income countries (LMICs), with only 5 of the 29 included studies (17%) conducted in LMICs, reducing the applicability of evidence to more resource-constrained settings where the shortage of health professionals is greatest. Of note, all 5 of the studies conducted in LMICs were published between 2014 and 2016, suggesting that the field of mLearning is developing in these countries. The studies included in this review covered a variety of areas within the medical, nursing, physiotherapy, and dental field; however, the diversity in the subjects taught, outcomes measured, and the inconsistent measurement tools used in the assessments, also need consideration when interpreting our findings.

The majority of studies focused on preregistration medical and nursing students and residents undertaking specialty training. A smaller number of studies focused on practicing physicians [32,35], practicing nurses [28], physiotherapy students [36] dental students [37], midwives and health extension workers [43]. There were no studies included involving pharmacists or occupational therapists and there was a lack of studies in general among allied health professionals indicating that mLearning interventions may not be implemented as commonly outside the field of medical and nursing education. More research is needed, especially in these fields, to assess whether mLearning is applicable more broadly across the health professions field.

As mLearning may involve the use of new technology, which can entail significant financial investment, the cost of such an introduction, especially in low-income settings, is a key factor when deciding to adopt new mediums for education. Unfortunately, only 2 studies, with conflicting results, performed an economic analysis comparing mLearning with traditional learning methods. As a result, there is limited evidence to draw any definitive conclusions on the costs and cost-effectiveness of mLearning. Similarly, there was little information on patient-related outcomes or changes in clinical behaviors/practices. A further aspect of mLearning that needs addressing is the impact of mLearning on the adverse/unintended effects of mLearning on patients as well as the learner.

For the majority of the studies, the intervention was introduced as supplementary learning to their standard education, and therefore questions remain as to how effective mLearning would be when introduced to a formal curriculum. In addition, the interventions were often introduced on an optional basis, where the use and intensity of the intervention were dependent on the individual and exact exposure and effect of the intervention are unknown.

Only 6 studies mentioned the use of learning theories to inform their instructional design. This indicates a major limitation of mLearning within health professions education. There is a lack of theoretical frameworks guiding effective instructional design so that learning pathways using adopted technologies could be delineated clearly [59,60]. To meet the future needs of the evolving landscape of health professions education, emphasis should be put on training health professionals who can fulfill these needs using mobile technologies. Therefore, the first step in these efforts is to carefully investigate how to use existing pedagogical frameworks to inform the design and development of mobile learning interventions that aim to achieve desired learning outcomes. In designing mLearning interventions, considerations of feasibility in real-life contexts, scalability and, sustainability over time are important for long term success [60].

### Strengths and Limitations of the Review

This review provides the most up-to-date evidence on the effectiveness of mLearning in health professions education and is supported by a comprehensive search strategy and the robust methodology that was applied at each stage of the screening, data extraction, and assessment of the evidence. This is illustrated by the larger body of evidence gathered in this review compared with previous reviews [18-21]. However, several biases may have been introduced in the review process.

Common biases include study eligibility criteria, identification and selection of studies, data extraction, and study appraisal. We tried to minimize or eliminate these biases in this review by adopting a variety of quality checks. We prespecified the eligibility criteria of the studies to be included in the review, and those were clearly defined in the protocol published before carrying out the review. This measure ensured that decisions on which studies were to be included were consistent and not based on characteristics of potentially eligible studies. The search strategy was devised and conducted by experienced librarians including all appropriate databases. As the search strategy was devised for a general project on digital education, it included Medical Subject Headings terms for several different digital education modalities. The search strategy retrieved a very large number of references. Titles and abstracts were screened independently by a team of reviewers and full-text inclusion assessment involved at least 2 reviewers. Furthermore, lead authors of the reviews of the other digital education modalities transferred studies from their reviews to other more appropriate reviews if necessary. Data availability bias may occur if some data are unavailable in the included studies and their unavailability is related to the study results. As with publication bias, this situation may lead to unrepresentative data and toward a false favorable effect. We contacted authors of studies with missing data or no data on specific outcomes to ask for those or to ask clarifications. Overall the risk of bias for most studies was judged to be high (because of a lack of information), with some instances of a high risk of bias for sequence generation, attrition, and reporting bias identified.

Reasons for downgrading the evidence included inconsistency, that is, high heterogeneity/differences in the direction of effect, with high the I^2^ values reported for each of the knowledge and skill comparisons. We did not identify a sufficient number of studies within the review comparisons to allow for the performance of subgroup analyses, which were prespecified in the protocol. With the number of studies of mLearning in health professions education continuing to rise over time, future reviews will be able to perform more focused subgroup analyses.

### Future Research

The review identified gaps in evidence, which if addressed, would provide more conclusive evidence on the effectiveness and cost-effectiveness of mLearning. Further research should do the following: assume validated and standardized outcome measures, use adequately powered trials, ensure that participants are adequately trained and empowered to use the mLearning devices, be adequately reported to allow independent replications, shift toward competency-based assessments, include theoretical underpinning in instructional design, include participants from other health professions such as pharmacy and occupational therapy; be conducted in more LMICs, incorporate a more in-depth study of the various aspects of mLearning (eg, interactivity, feedback) and how each specific component affects study outcomes, provide information about the effects of mLearning on patient outcomes, provide information on cost and cost-effectiveness of mLearning, provide information on potential unintended effects of mLearning, and include both short-term and retention (follow up) outcome data.

### Conclusions

mLearning is a novel educational strategy that is rapidly developing in the field of health professions education. The synthesis of data in this review shows that mobile learning is at least as or potentially more effective than traditional learning. However, the effectiveness of mLearning in health professions education is not certain because of the lack of validated and standardized outcome measures, and heterogeneity between both interventions and outcome assessments. Furthermore, there is a need for research to expand to the realm of cost-effectiveness, to fully understand the value of mLearning in health professions education. Further research is necessary to conclusively evaluate the effectiveness and cost-effectiveness of mLearning.

## Appendix

Multimedia Appendix 1 MEDLINE (Ovid) search strategy.

Multimedia Appendix 2 Data extraction form.

Multimedia Appendix 3 Characteristics of included studies.

Multimedia Appendix 4 Summary of findings table for mLearning versus traditional learning.

Multimedia Appendix 5 Summary of findings table for blended learning vs traditional learning.

Multimedia Appendix 6 Risk of bias graph.

Multimedia Appendix 7 Risk of bias for cluster randomized controlled trials.

## Abbreviations

GAT: Geriatric Assessment Tool
GPRS: general packet radio services
GRS: global rating scale
GSM: global system for mobile communications
LMIC: low- and middle-income countries
MP3: Moving Picture Experts Group-1 audio layer 3
MCQ: multiple choice questionnaire
PDA: personal digital assistant
RCT: randomized controlled trial
SMD: standardized mean difference
SMS: short message service
