# Supplementary material for: Mobile Digital Education for Health Professions: Systematic Review and Meta-Analysis by the Digital Health Education Collaboration
Source: J Med Internet Res. 2019 Feb 12;21(2):e12937. doi: 10.2196/12937 (PMC6390189; doi:10.2196/12937)
Supplement: Multimedia Appendix 2 [file jmir_v21i2e12937_app2.docx]

|  | Study ID |  |
| --- | --- | --- |
| **2. Study Identification** | 2.1. Journal where the study was published |  |
|  | 2.2. Type of publication |  |
|  | 2.3. Authors' affiliation |  |
| **3. Study design** | 3.1. Study design as specified in the report |  |
|  | 3.2. Study aims & objectives |  |
|  | 3.3. Countries where the study was conducted |  |
|  | 3.4. WHO region |  |
|  | 3.5.World Bank income category |  |
|  | 3.6. Study start date |  |
|  | 3.7. Study end date |  |
|  | 3.8. Method of comparison |  |
| **4. Participants** | 4.1. Total number of participants invited to take part in the study |  |
|  | 4.2. Total number of participants who agreed to take part in the study |  |
|  | 4.3. Total number of participants meeting the inclusion criteria for participation in the study |  |
|  | 4.4. Total number of participants included in the study |  |
|  | 4.5. If cluster RCT, total number of clusters initially included in the study |  |
|  | 4.6. If cluster RCT, total number of clusters randomised |  |
|  | 4.7. Inclusion criteria |  |
|  | 4.8. Exclusion criteria |  |
| **5. Intervention & Comparison** | 5.1. Total number of experimental groups (including the control group) |  |
|  | 5.2. Were groups tested for baseline differences? |  |
|  | 5.2.1. If there were baseline differences, please specify what the difference was |  |
|  | 5.3. Indicate the type of degree or qualification that participants were pursuing |  |
|  | If other, please specify: |  |
|  | 5.4. Year of study within the anticipated degree or qualification |  |
|  | **5.5. Control group** |  |
|  | 5.5.1. Total number of participants/clusters allocated to the control group |  |
|  | 5.5.2. Mean age (standard deviation) of the participants in the control group |  |
|  | 5.5.3. Name of educational intervention used as control |  |
|  | 5.5.4. Description of the control condition |  |
|  | 5.5.5. Field of study |  |
|  | 5.6.6. Exposure to the control condition during the whole study |  |
|  | 5.5.7. Total exposure time to the intervention |  |
|  | 5.5.8. Type of technology/devices used to deliver the intervention |  |
|  | 5.5.9. Delivery approach of the intervention |  |
|  | *If other, please specify:* |  |
|  | 5.5.10. Was the usual delivery mode of the assessment changed? |  |
|  | 5.5.11. If yes, please specify |  |
|  | 5.5.12. Was the delivery mode of the assessment uniform across all the experimental groups? |  |
|  | **5.6. Intervention group I** |  |
|  | 5.6.1. Total number of participants/clusters allocated to this intervention group. |  |
|  | 5.6.2. Mean age (standard deviation) of the participants in this intervention group |  |
|  | 5.6.3. Name of educational intervention used in this intervention group |  |
|  | 5.6.4. Description of this intervention condition |  |
|  | 5.6.5. Field of study |  |
|  | 5.6.6. Exposure to this intervention condition during the whole study |  |
|  | 5.6.7. Total exposure time to the intervention |  |
|  | 5.6.8. Type of technology/devices used to deliver the intervention |  |
|  | 5.6.9. Delivery approach of the intervention |  |
|  | *If other, please specify:* |  |
|  | 5.6.10. Was the usual delivery mode of the assessment changed? |  |
|  | 5.6.11. If yes, please specify |  |
|  | 5.6.12. Was the delivery mode of the assessment uniform across all the experimental groups? |  |
| **6. Outcomes** | 6.1. Was 'Knowledge' measured? - If not, please go to section 6.2. |  |
|  | 6.1.1. Instrument or measure used to asses knowledge - as specified by the study authors |  |
|  | 6.1.2. Is this a validated instrument? |  |
|  | 6.2. Were 'Skills' measured? - If not, please go to section 6.3. |  |
|  | 6.2.1. Instrument or measure used to asses skills - as specified by the study authors |  |
|  | 6.2.2. Is this a validated instrument? |  |
|  | 6.3. Were 'Attitudes' measured? - If not, please go to section 6.4. |  |
|  | 6.3.1. Instrument or measure used to asses attitudes - as specified by the study authors |  |
|  | 6.3.2. Is this a validated instrument? |  |
|  | 6.4. Was 'Student satisfaction' measured? - If not, please go to section 6.5. |  |
|  | 6.4.1. Instrument or measure used to asses student satisfaction - as specified by the study authors |  |
|  | 6.4.2. Is this a validated instrument? |  |
|  | 6.5. Was an economic evaluation of the eLearning intervention performed? |  |
|  | 6.5.1. Were quantitative indicators like costs, investments, hardware, software, license fees and benefits/savings of the eLearning intervention measured? |  |
|  | 6.5.2. Was the urgency of the eLearning intervention (i.e., due to a new regulation or organisational demand) mentioned? |  |
|  | 6.5.3. Were qualitative-strategic indicators of the eLearning intervention like quality and performance improvements measured? |  |
|  | 6.5.4. Were external factors of the eLearning intervention like synergy effects or economies of scope measured? |  |
|  | 6.5.5. Please list any additional economic indicators that were measured |  |
| **9. Study findings** | **9.1. Control group** |  |
|  | 9.1.1. Outcome reported |  |
|  | 9.1.2. Measure of effect size (as measured by the study authors) |  |
|  | 9.1.3. Measure of dispersion (as measured by the study authors) |  |
|  | 9.1.1. Outcome reported |  |
|  | 9.1.2. Measure of effect size (as measured by the study authors) |  |
|  | 9.1.3. Measure of dispersion (as measured by the study authors) |  |
|  | 9.1.1. Outcome reported |  |
|  | 9.1.2. Measure of effect size (as measured by the study authors) |  |
|  | 9.1.3. Measure of dispersion (as measured by the study authors) |  |
|  | 9.1.1. Outcome reported |  |
|  | 9.1.2. Measure of effect size (as measured by the study authors) |  |
|  | 9.1.3. Measure of dispersion (as measured by the study authors) |  |
|  | If more than one outcome was reported, please insert more cells here and copy and paste the relevant data entry boxes. |  |
|  | **9.2. Intervention I group** |  |
|  | 9.2.1. Outcome reported |  |
|  | 9.2.2. Measure of effect size (as measured by the study authors) |  |
|  | 9.2.3. Measure of dispersion (as measured by the study authors) |  |
|  | 9.2.1. Outcome reported |  |
|  | 9.2.2. Measure of effect size (as measured by the study authors) |  |
|  | 9.2.3. Measure of dispersion (as measured by the study authors) |  |
|  | 9.2.1. Outcome reported |  |
|  | 9.2.2. Measure of effect size (as measured by the study authors) |  |
|  | 9.2.3. Measure of dispersion (as measured by the study authors) |  |
|  | 9.2.1. Outcome reported |  |
|  | 9.2.2. Measure of effect size (as measured by the study authors) |  |
|  | 9.2.3. Measure of dispersion (as measured by the study authors) |  |
|  | If more than one outcome was reported, please insert more cells here and copy and paste the relevant data entry boxes. |  |
|  | **9.5. Comparison I** |  |
|  | 9.5.1. Please indicate the intervention groups being compared |  |
|  | 9.5.2. Please indicate the outcomes being compared |  |
|  | 9.5.3. Statistical test used for the comparison |  |
|  | 9.5.4. Result of the test |  |
|  | 9.5.5. P value / Confidence intervals |  |
| **10. ImplementatioNAdoption of eLearning interventions** | 10.1. Organizational setting |  |
|  | 10.2. Technological infrastructure |  |
|  | 10.3. Instructional Systems Design and Curriculum development |  |
|  | 10.4. Delivery |  |
|  | 10.5. Advantages of eLearning - as reported by the study authors |  |
|  | 10.6. Disadvantages of eLearning - as reported by the study authors |  |
| **11. Sustainability of eLearning interventions** | 11.1. Source of financing - as reported by the study authors |  |
|  | 11.2. Did the intervention undergo a formal accreditation process within the host institution? |  |
|  | 11.3. If yes, please describe |  |
|  | 11.4. Was the eLearning intervention developed for this study consequently adopted as a formal method for the delivery of education at the host institution? |  |
|  | 11.5. If yes, please specify |  |
| **12. Miscellanous** | 12.1. Study conclusions - as stated by the study authors |  |
|  | 12.2. Limitations of the study - as reported by the study authors |  |
|  | 12.3. Was contact with the study authors sought? - If No, please go to section 12.5 |  |
|  | 12.4. Please indicate the nature of the information requested from the study authors |  |
|  | 12.5. Please indicate the results of the request for information |  |
|  | 12.6. Additional notes |  |
