# Supplementary material for: Mobile Digital Education for Health Professions: Systematic Review and Meta-Analysis by the Digital Health Education Collaboration
Source: J Med Internet Res. 2019 Feb 12;21(2):e12937. doi: 10.2196/12937 (PMC6390189; doi:10.2196/12937)
Supplement: Multimedia Appendix 3 [file jmir_v21i2e12937_app3.docx]

Multimedia Appendix 3. Characteristics of included studies

| **Study ID (Author Year), Reference, country** | **Setting** | **Intervention** | **Control** | **Outcome and measurement instrument** |
| --- | --- | --- | --- | --- |
|  | **Population (n)** | mLearning device |  |  |
|  | **Field of study** | Content |  |  |
|  |  | Frequency and duration |  |  |
| Alam (2016)[27], Canada | University | Tablet / smartphone | A narrated PPT on a podcast | 1) Knowledge  with an MCQ (unspecified no. of items)  2) Skills with a Direct Observation of Procedural Skills |
|  | Medical students(63) | A narrated PPT on a podcast with video demonstrations; and guided mental practice. |  |  |
|  | Anesthesia: airway management | Once, duration not specified. |  |  |
| Alipour (2014)[28], Iran | Hospital | Basic mobile phone | Traditional learning | 1) Knowledge with an MCQ (10 items) |
|  | Nurses(60) | SMS text messages |  |  |
|  | Breast cancer screening | Three or four SMSs per day for 17 days. |  |  |
| Amer (2017)[29], US | University | Tablet / smartphone | A video lecture with slides | 1) Knowledge  with an MCQ (21 items) |
|  | Medical students(100) | “Carpal Tunnel Surgery” module |  |  |
|  | Carpal tunnel surgical knowledge | The module was completed and repeated three times in one sitting. |  |  |
| Baumgart (2017)[30], Germany | Hospital | Tablet / smartphone | Traditional learning | 1) Knowledge  with the Medical Knowledge Self-Assessment Program MKSAP® |
|  | Medical students and residents(80) | Access to online resources |  |  |
|  | General medical education | Participant’s received device for four months, use was dependent on the individual. |  |  |
| Bochicchio (2006)[55], US | Hospital | PDA | Traditional learning | 1) Knowledge  with an MCQ (50 items) |
|  | Trauma and critical care fellows(12) | Johns Hopkins Antibiotic Guide |  |  |
|  | Infectious diseases management | Participant’s received device for six months, use was dependent on the individual. |  |  |
| Calhoun (2017)[31], US | Hospital | Tablet / smartphone | Traditional learning | 1) Skill with the Gap-Kalamazoo Communication  Skills Assessment Form (GKCSAF) |
|  | Emergency medicine service providers(148) | An app with didactic modules and video simulation recordings. |  |  |
|  | Communication skills | One time for two hours |  |  |
| Chen (2014)[32], China | Health care center | Basic mobile phone | Traditional learning | 1) Knowledge with an MCQ (10 items)  2) Behavior  with changes in prescribing practices |
|  | Family Physicians (977) | SMS text messages |  |  |
|  | Managing infections (upper respiratory tract and middle ear) | Three messages a week for six weeks |  |  |
| Chuang (2013)[33], Taiwan | University | Basic mobile phone | Traditional learning | 1) Knowledge with an MCQ (20 items)  2) Satisfaction with Likert scale (10 items) (0-5 scale) |
|  | Nursing students(111) | Lectures + SMS text messages |  |  |
|  | Cardiovascular medication | Twice a day for 10 days |  |  |
| Choi (2014)[52], South Korea | University | Tablet / smartphone | Traditional learning | 1) Skill with the Global Interpersonal Communication Competence Scale (GICC) |
|  | Nursing students(87) | Lectures, video clip group presentations feedback and sharing |  |  |
|  | Communication skills | Participants received device for 8 weeks, use was dependent on the individual. |  |  |
| De Oliveira (2013)[34], US | University | Tablet / smartphone | Traditional learning | 1) Skill with Direct Observation of Procedural Skills |
|  | Medical students(20) | iLarynx app + lecture |  |  |
|  | Surgical skills: upper airway anatomy and fiberoptic intubation | One time for 30 minutes |  |  |
| Donato (2014)[35], US | Hospital | MP3 player | Traditional learning | 1) Skill with an MCQ (20 items)  2) Attitude with Likert scale (1 item) (1-6 scale)  3) Satisfaction  with Likert scale (1 item) (1-6 scale) |
|  | Physicians and residents(87) | Heart sound audio files |  |  |
|  | Auscultation | Participants received device for three months, use was dependent on the individual. |  |  |
| Fernandez-Lao (2016)[36], Spain | University | Tablet / smartphone | Traditional learning | 1) Knowledge with an MCQ (20 items)  2) Skill with Direct Observation of Procedural Skills  3) Satisfaction with Likert scale (1 item) (0-10 scale) |
|  | Physiotherapy students (49) | Lecture + a mobile app with multi-media content for self-study. |  |  |
|  | Palpation and ultrasound imaging skills | Participants received device for two weeks, use was dependent on the individual. |  |  |
| Gadbury-Amyot (2016)[37], US | University | Tablet / smartphone | Traditional learning | 1) Skill with a practical assessment  2) Attitude with Likert scale (4 items) (1-5 scale) |
|  | Dental students(188) | Videos |  |  |
|  | Dental procedures | Two hours per week for one semester |  |  |
| Hansen (2011)[38], New Zealand | University | iPod | Traditional learning | 1) Skill with Direct Observation of Procedural Skills  2) Attitude with a survey |
|  | Medical students(21) | Video + clinical skills course |  |  |
|  | Surgical skills: urinary catheterization | Participants received device for three months, use was dependent on the individual. |  |  |
| John (2015)[39], UK | Hospital | Tablet / smartphone | Traditional learning | 1) Skill with Direct Observation of Procedural Skills |
|  | Neurosurgery Trainees(20) | Lecture + “VCath” application |  |  |
|  | Surgical skills: Neurosurgery - performing ventriculostomy | One time for 20 minutes |  |  |
| Johnston (2010)[40], US | University | iPod | Traditional learning | 1) Knowledge  with an MCQ (unspecified no. of items) |
|  | Nursing students(35) | Pre-recorded lectures in iPods and optional lectures |  |  |
|  | Medical-surgical nursing | Participants received device for one semester, use dependent on individual. |  |  |
| Kim (2017)[41], South Korea | University | Tablet / smartphone | Traditional learning | 1) Knowledge with an MCQ (8 items) + 6 true and false questions  2) Attitude with Likert scale (11 items) (1-5 scale)  3) Satisfaction  with Likert scale (8 items) (1-5 scale) |
|  | Nursing students(80) | “Caring for Infants with Airway Obstruction” application |  |  |
|  | Infant airway obstruction | Participants received device for one month, use was dependent on the individual. |  |  |
| Kucuk (2016)[42], Turkey | University | Tablet / smartphone | Traditional learning | 1) Knowledge with an MCQ (30 items) |
|  | Medical students(70) | An app which students used while looking at pages of a magic book to see multimedia materials appearing on the mobile device screen |  |  |
|  | Anatomy: Neuroanatomy | Five course hours along with participants receiving devices for an unspecified period of time after class, use was dependent on the individual. |  |  |
| Lund (2016)[43], Ethiopia | Health care facilities | Tablet / smartphone | Usual learning | 1) Patient-related outcomes: Perinatal mortality + stillbirth rates  2) Knowledge with a questionnaire  3) Skill with Direct Observation of Procedural Skills |
|  | Health care workers(176) | “Safe delivery app” |  |  |
|  | Neonatal resuscitation | Participants received device for 1 year, use was dependent on the individual. |  |  |
| Mandry (2013)[44], US | Hospital | Basic mobile phone | Traditional learning | 1) Knowledge with the SAEM National EM M4 Exam Version |
|  | Medical students (65) | SMS text messages |  |  |
|  | Emergency medicine | Participants received an unspecified no. of SMSs for two or four weeks |  |  |
| Martinez (2017) [45], Chile | University | Tablet / smartphone | Usual learning | 1) Knowledge  with an MCQ (90 items) |
|  | Medical students(80) | Smartphone application |  |  |
|  | Internal medicine | Participants had access to the application for one month, use was dependent on the individual. |  |  |
| McLeod,(2009)[46], US | Hospital | PDA | PDA with no additional program | 1) Knowledge  with a Questionnaire (10 item) |
|  | Residents: internal medicine(38) | Lecture modules + a PDA program. |  |  |
|  | Geriatrics | Participants received device for one month, use was dependent on the individual. |  |  |
| Mount (2015)[47], US | Hospital | Basic mobile phone | Usual learning | 1) Knowledge  with a MCQ (30 items) |
|  | Residents: Family medicine (269) | SMS text messages |  |  |
|  | Musculoskeletal medicine | Six messages a week 12 weeks |  |  |
| Ng (2015)[48], Singapore | University | Tablet / smartphone | Traditional learning | 1) Skill with a timed quiz  2) Attitude with Likert scale (3 items) (0-3 scale) |
|  | Medical students(72) | 3D model on iPad + text books |  |  |
|  | Anatomy: Epitympanum | One time for 30 minutes |  |  |
| Nilsson (2017)[49], Denmark | Hospital | Tablet / smartphone | Traditional learning | 1) Knowledge with a theoretical test  2) Skill with Direct Observation of Procedural Skills  3) Cost-effectiveness  with the Program Effectiveness and Cost Generalization model |
|  | Residents(38) | App-guided training |  |  |
|  | Ultrasound skills | One time for two hours |  |  |
| O'Donovan (2016)[50], Kenya | University | Tablet / smartphone | Traditional learning | 1) Skill with Direct Observation of Procedural Skills |
|  | Medical students(51) | Multi-media content + usual clinical teaching |  |  |
|  | Cardiovascular and abdominal clinical examinations | Participants received device for three weeks, use was dependent on the individual. |  |  |
| Prasad (2016)[51], US | Hospital | Tablet / smartphone | Traditional learning | 1) Knowledge  with a method that was not clearly reported |
|  | Residents(118) | Lecture videos |  |  |
|  | Cardiovascular medical knowledge | Received device every second month for one year, use was dependent on the individual. |  |  |
| Tempelhof (2009)[53], US | Hospital | iPod | Traditional learning | 1) Knowledge with an MCQ (25 items) |
|  | Residents(30) | Narrated PPT slides from conferences |  |  |
|  | Patient care | Received device for 1 month, use was dependent on the individual. |  |  |
| Walter (2014), [54], US | Hospital | Tablet / smartphone | Traditional learning | 1) Knowledge with a Questionnaire (25 items) |
|  | Residents and interns (30) | A daily email and/or RSS feed prompt to their mobile device with 5 questions + lectures |  |  |
|  | Critical care medicine | Participants had access to the application for 4 weeks, use was dependent on the individual. |  |  |

Abbreviations: N = Number of participants, MCQ = Multiple Choice Questions, PDA = Personal Digital Assistant, PPT = PowerPoint Presentation, RSS = Rich Site Summary, SMS = Short Message Service, UK = United Kingdom, US = United States of America, 3D = three dimensional
