# Supplementary material for: Mobile Digital Education for Health Professions: Systematic Review and Meta-Analysis by the Digital Health Education Collaboration
Source: J Med Internet Res. 2019 Feb 12;21(2):e12937. doi: 10.2196/12937 (PMC6390189; doi:10.2196/12937)
Supplement: Multimedia Appendix 4 [file jmir_v21i2e12937_app4.docx]

Multimedia Appendix 4. Summary of findings table for blended learning vs traditional learning

| Blended learning compared with traditional learning | | | | | |
| --- | --- | --- | --- | --- | --- |
| Population: Pre- and Post-registration healthcare professionals  Settings: Universities and Hospitals  Intervention: Blended learning (mLearning plus traditional learning)  Comparison: Traditional learning | | | | | |
| Outcomes | Illustrative comparative risks* (95% CI) | Relative effect (95% CI) | No of Participants (studies) | Quality of the evidence (GRADE) | Comments |
| Knowledge  Assessed with MCQ, questionnaires (immediately post-intervention) | The mean score in the intervention group was 0.20 standard deviations higher (-0.47 lower to 0.86 higher) than the mean score in the traditional learning group. | Not estimable | 345 participants (6 studies) | ⊕⊕⊝⊝  low^a,b^ | The standard deviations was derived from a SMD of 0.20 (95% CI: -0.47 to 0.86) which indicates little or no difference between groups. |
| Skills  Assessed with checklists, timed quiz, Objective Structured Assessments of Procedural Skills, (immediately post-intervention) | The mean score in the intervention group was 1.06 standard deviations higher (0.09 to 2.03 higher) than the mean score in the traditional learning group. | Not estimable | 321 participants (7 studies) | ⊕⊕⊝⊝ low^a,b^ | The standard deviations was derived from a SMD of 1.06 (95% CI: 0.09 to 2.03) which indicates a large effect size. The results of a further study (183 participants) were not pooled due to incomplete data. |
| Attitude  Assessed with Likert scale (immediately post-intervention) | The mean score in the intervention group was 0.78 standard deviations higher (-0.11 lower to 1.68 higher) than the mean score in the traditional learning group. | Not estimable | 21 participants (1 study) | ⊕⊝⊝⊝ very low^a,b,d^ | The standard deviations was derived from a SMD of 0.78 (95% CI: -0.11 to 1.68) which indicates little or no difference between groups. The results of two studies [37, 48] (256 participants) were not pooled due to incomplete data. |
| Satisfaction | Not estimable | Not estimable | 0 participants (0 studies) | Not estimable | No study assessed participants' post intervention satisfaction scores in both the blended learning intervention group and the traditional learning group. Two studies [33, 36] assessed participants' post intervention satisfaction in the intervention group only, hence, we were unable to judge the effect of the interventions due to missing or incomparable outcome data. |
| CI: Confidence interval; RR: Risk Ratio; MCQ: Multiple choice questions, NA: Not Applicable | | | | | |
| GRADE Working Group grades of evidence High quality: Further research is very unlikely to change our confidence in the estimate of effect. Moderate quality: Further research is likely to have an important impact on our confidence in the estimate of effect and may change the estimate. Low quality: Further research is very likely to have an important impact on our confidence in the estimate of effect and is likely to change the estimate. Very low quality: We are very uncertain about the estimate. | | | | | |

Footnotes

^a^ Rated down by one level for study limitations: the risk of bias was unclear for sequence generation and allocation concealment in majority of the studies.

^b^ Rated down by one level for inconsistency: the heterogeneity is high with large variations in effect and lack of overlap among confidence intervals (CIs).

^c^ Rated down by one level for imprecision: number of participants (effective sample size) in many studies is less than the number of patients generated by a conventional sample size calculation for a single adequately powered trial (optimal information size)

^d^ Downgraded as results were obtained from single small study
