# Supplementary material for: Mobile Digital Education for Health Professions: Systematic Review and Meta-Analysis by the Digital Health Education Collaboration
Source: J Med Internet Res. 2019 Feb 12;21(2):e12937. doi: 10.2196/12937 (PMC6390189; doi:10.2196/12937)
Supplement: Multimedia Appendix 5 [file jmir_v21i2e12937_app5.docx]

Multimedia Appendix 5. Summary of findings table for mLearning vs traditional learning

| mLearning compared with traditional learning | | | | | |
| --- | --- | --- | --- | --- | --- |
| Population: Pre- and Post-registration healthcare professionals  Settings: Universities and Hospitals  Intervention: mLearning  Comparison: Traditional learning | | | | | |
| **Outcomes** | **Illustrative comparative risks* (95% CI)** | **Relative effect (95% CI)** | **No of Participants (studies)** | **Quality of the evidence (GRADE)** | **Comments** |
| **Knowledge**  Assessed with MCQ, questionnaires, theoretical test (from post-intervention to 4-6 weeks follow-up) | The mean score in the intervention group was 0.43 standard deviations higher (0.05 to 0.80 higher) than the mean score in the traditional learning group. | Not estimable | 1828 participants (11 studies) | ⊕⊕⊝⊝ low^a,b^ | The standard deviations was derived from a SMD of 0.43 (95% CI: 0.05 to 0.80) which indicates a small effect size. |
| **Skills**  Assessed with checklists, MCQ, Objective Structured Assessments of Procedural Skills, (immediately post-intervention) | The mean score in the intervention group was 1.12 standard deviations higher (0.56 to 1.69 higher) than the mean score in the traditional learning group. | Not estimable | 529 participants (5 studies) | ⊕⊕⊕⊝ moderate^a^ | The standard deviations was derived from a SMD of 1.12 (95% CI: 0.56 to 1.69) which indicates a large effect size. |
| **Attitude**  Assessed with Likert scale (immediately post-intervention) | The mean score in the intervention group was 0.51 standard deviations higher (0.20 to 0.81 higher) than the mean score in the traditional learning group. | Not estimable | 167 participants (2 studies) | ⊕⊝⊝⊝ low^a,c^ | The standard deviations was derived from a SMD of 0.51 (95% CI: 0.20 to 0.81) which indicates a moderate effect size. |
| **Satisfaction**  Assessed with Likert scales (immediately post-intervention) | The mean score in the intervention group was 0.39 standard deviations higher (-0.29 lower to 1.06 higher) than the mean score in the traditional learning group. | Not estimable | 167 participants (2 studies) | ⊕⊝⊝⊝ very low^a,b,c^ | The standard deviations was derived from a SMD of 0.39 (95% CI: -0.29 to 1.06) which indicates little or no difference between groups. |
| CI: Confidence interval; RR: Risk Ratio; MCQ: Multiple choice questions, NA: Not Applicable | | | | | |
| GRADE Working Group grades of evidence High quality: Further research is very unlikely to change our confidence in the estimate of effect. Moderate quality: Further research is likely to have an important impact on our confidence in the estimate of effect and may change the estimate. Low quality: Further research is very likely to have an important impact on our confidence in the estimate of effect and is likely to change the estimate. Very low quality: We are very uncertain about the estimate. | | | | | |

Footnotes

^a^ Rated down by one level for study limitations: the risk of bias was unclear for sequence generation and allocation concealment in majority of the studies.

^b^ Rated down by one level for inconsistency: the heterogeneity is high with large variations in effect and lack of overlap among confidence intervals (CIs).

^c^ Rated down by one level for imprecision: number of participants (effective sample size) in many studies is less than the number of patients generated by a conventional sample size calculation for a single adequately powered trial (optimal information size)
