# Supplementary figures and images for: Mobile Digital Education for Health Professions: Systematic Review and Meta-Analysis by the Digital Health Education Collaboration
Source: J Med Internet Res. 2019 Feb 12;21(2):e12937. doi: 10.2196/12937 (PMC6390189; doi:10.2196/12937)

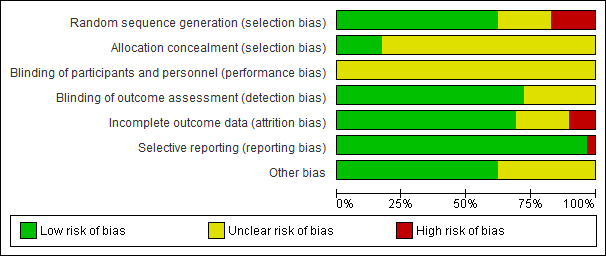

Supplement: Multimedia Appendix 6 [file jmir_v21i2e12937_app6.png]
