# Supplementary material for: Mobile Digital Education for Health Professions: Systematic Review and Meta-Analysis by the Digital Health Education Collaboration
Source: J Med Internet Res. 2019 Feb 12;21(2):e12937. doi: 10.2196/12937 (PMC6390189; doi:10.2196/12937)
Supplement: Multimedia Appendix 7 [file jmir_v21i2e12937_app7.docx]

**Multimedia appendix 2: Risk of Bias for Cluster RCTs**

| Study ID | Recruitment bias | Baseline imbalance | Loss of clusters | Incorrect analysis | Comparability with individual trials |
| --- | --- | --- | --- | --- | --- |
| Calhoun 2017 | Unclear | Low | Low | Low | Low |
|  | Comment: Recruitment and sequence generation was not described clearly. Insufficient evidence to make a judgement. | Comment: There were no statistically significant differences between the two groups. | Comment: No clusters were lost. | Comment: As described in our methods section, we corrected for potential cluster effects using the outcome data provided in the study and the direction and effect of the interventions remained the same. | Comment: The results from this study are in line with those with individual trials. |
| Chen 2014 | Low | Low | Low | Low | Low |
|  | Quote (report): "We used the health centre as the unit of randomization. A cluster design was used to avoid biases arising from the possible conveyance of information by members of the intervention group to members of the control group if both were located at the same health centre. Randomization was done in two stages. First, with the help of the health administration department of Gansu province, we sent invitation letters to all 1333 health centres in Gansu province. By the deadline, 163 health centres had agreed to participate in our study. From these centres we randomly selected 100 for inclusion in the trial; we then used a computer-generated random sequence to select the clusters for intervention." | Comment: There were no statistically significant differences between the two groups. | Comment: No clusters were lost. | Table 2 shows the cluster and individual level information are compared and presented (clusters are considered into random effect). Therefore, we can say that this study addressed the clustering effect. | Comment: The results from this study are in line with those with individual trials. |
| Lund 2016 | Low | Low | Low | Low | Low |
|  | Quote (report): "a computer-generated random number table"  "Facilities were randomized rather than individuals to avoid contamination among health careworkers in the same facility, such as health care workers showing the application and animation videos to the control group."  No evidence of recruitment bias. | Comment: There were no statistically significant differences between the two groups. | Comment: No clusters were lost. | Comment: As described in our methods section, we corrected for potential cluster effects using the outcome data provided in the study and the direction and effect of the interventions remained the same, | Comment: The results from this study are in line with those with individual trials. |
| Mount 2015 | Low | Low | Low | Low | Low |
|  | Quote (report): "Program names were written on folded cards then drawn out of the group and placed alternately in two piles. A coin flip determined which group became intervention and control. Randomization at the institutional level was chosen over the individual level to avoid contamination by residents sharing or discussing texts with those in the control group." | Comment: There were no statistically significant differences between the two groups. | Comment: No clusters were lost. | Comment: Generalized Linear Model and the Generalized Estimating Equation were used in this study. As described in our methods section, we corrected for potential cluster effects using the outcome data provided in the study and the direction and effect of the interventions remained the same. | Comment: The results from this study are in line with those with individual trials. |
